# Supplementary material for: Occupational exposure to HIV among nurses at a major tertiary hospital: Reporting and utilization of post-exposure prophylaxis; A cross-sectional study in the Western Cape, South Africa
Source: PLoS One. 2020 Apr 14;15(4):e0230075. doi: 10.1371/journal.pone.0230075 (PMC7156052; doi:10.1371/journal.pone.0230075)
Supplement: S1 Doc — (PDF) [file pone.0230075.s001.pdf]


QUESTIONNAIRE

Occupational exposure to HIV among nurses at Tygerberg Hospital, Cape Town: Reporting and utilization of post exposure prophylaxis; A cross-sectional study.

INSTRUCTIONS

Kindly fill in the questionnaire by choosing the number (1 or 2 or 3 etc.) that corresponds to your answer. Use either a tick (√) or circle out your choice. For structured questions, kindly please give a brief answer. Should you make a mistake, please cancel out the incorrect option and select the preferred choice.

Section 1: Sociodemographic characteristics

1a. What is your gender?	Male	1
	Female	2
1b. Which race do you identify yourself with?	Black	1
	White	2
	Coloured	3
	Other	4
1c. Marital status	Single	1
	Married	2
	Divorced	3
	Widowed	4
1d.What was your age at your last birthday?	______________________	
1e. How many years have you been practising as a nurse, since you qualified?	______________________	
1f. Which department/ward do you work?	______________________	
1g. What is your highest level of education?	Diploma/certificate	1
	Bachelor’s degree	2
	Master’s degree	3
	Other	5
1h. Have you ever attended any formal training on HIV PEP?	Yes	1

Study number	

	No	2

Section 2: Frequency of occupational HIV exposure during the last 12 months

In this section kindly answer questions about whether you have been exposed to HIV either through injury such as needle sticks or through exposure to body fluids during your work in the hospital. These questions only relate to the period from 01 January 2018 to the period 30 January 2019 (one year).

2a. Have you ever been exposed to any infectious material either through exposure to body fluids or through a needle stick injury in the work place in the past 12 months?<br><br>*IF THE ANSWER IS NO, PLEASE PROCEED TO SECTION 3 OF THE QUESTIONNAIRE*	Yes	1
	No	2
	Don't remember	3
2b. If yes, when did this exposure happen? (state date if possible)	______________	
2c. If you can't remember when exactly it happened, roughly how many months ago did this happen?	______________	
2d. If it happened more than once, how many times did it happen?	______________	
2e. If you were exposed, which of the following were you exposed to? (Choose all that apply)	Needle stick injury	1
	Cut by a sharp object	2
	Any contact with body fluids including blood on mucosal surfaces	3
2f. Did you seek HIV post exposure prophylaxis (PEP) treatment after exposure?	Yes	1
	No	2
2f.i. If yes, did you start on HIV PEP treatment	Yes	1
	No	2
2g. If yes, did you finish the treatment?	Yes	1
	No	2
2h. If no, please give the reason for discontinuing the treatment.	Experiencing side effects	1
	Assuming that it was enough	2
	Found out that HIV status of person was negative	3

Study number	

2i. How long after the exposure did you report it to the relevant persons?	Immediately	1
	A few hours later	2
	A few days later	3
2j. If the exposure was not reported please give a reason from the options below on why you could not. Please choose all that may apply.<br>Being too busy at the time of exposure		1
The sharps that caused the injury was never used on a patient		2
Sharp object used on patient but was not disease of concern		3
Did not know where to should report the incident		4
Did not know how to report the incident		5
Colleagues told me not to worry		6

Section 3. Knowledge on HIV post exposure prophylaxis (PEP)

In this section, I will kindly need you to answer questions on what you know about HIV PEP.

3a. Have you ever heard about HIV PEP?	Yes	1
	No	2
3b. If yes, from what source did you get the information? (Choose all that apply)	Training	1
	Mass media	2
	Friends	3
	Journals	4
	Internet	5
	Other	6
	I have never heard of PEP	
3c. Is there a PEP protocol/ guideline in your work area?	Yes	1
	No	2
	I don't know	3
3d. When do you think HIV PEP should NOT be administered if exposed?<br>When source patient is HIV negative		1
When the patient is known to be HIV positive		2
When the HIV status of the source is unknown		3
I don't know		4
3e. When is the *recommended* time to take PEP		
Anytime after exposure		1
Within 72 hours of exposure		2
I don't know		3
3f. Under which circumstance would you NOT take PEP?<br>Exposure to blood and blood products from known HIV positive patients		1
Exposure to infectious material from patient whose HIV status is unknown		2

Study number	

Contact with patient body fluids which do not pose risk e.g. tears, non-blood stained saliva, urine		3
3. What is the length of time for taking HIV PEP?	For 28 days	1
	For six moths	2
	For life time	3
	I don't know	4

Section 4: Attitude towards HIV PEP

In this section we would like to know about your attitude towards HIV PEP. Please indicate whether you strongly agree or agree or disagree or strongly disagree or whether you are neutral.

**4a. Do you think HIV PEP is Important?**	**Strongly agree**	**1**
	**Agree**	**2**
	**Neutral**	**3**
	**Disagree**	**4**
	**Strongly disagree**	**5**
	**I don't know**	**6**
**4b. Do you believe that training on HIV PEP is important to influence people to comply to PEP guidelines?**	**Strongly agree**	**1**
	**Agree**	**2**
	**Neutral**	**3**
	**Disagree**	**4**
	**Strongly disagree**	**5**
	**I don't know**	**6**
**4c. Do you think there should be an HIV PEP guideline poster on the walls of your working area?**	**Strongly agree**	**1**
	**Agree**	**2**
	**Neutral**	**3**
	**Disagree**	**4**
	**Strongly disagree**	**5**
	**I don't know**	**6**
**4d. Do you think PEP reduces likelihood of being HIV positive after exposure?**	**Strongly agree**	**1**
	**Agree**	**2**
	**Neutral**	**3**
	**Disagree**	**4**
	**Strongly disagree**	**5**
	**I don't know**	
**4e. Do you think that HIV PEP should be administered if the exposure is not with patient of known HIV positive status?**	**Strongly agree**	**1**
	**Agree**	**2**
	**Neutral**	**3**
	**Disagree**	**4**
	**Strongly disagree**	**5**
	**I don't know**	**6**
**4f. What is your opinion on the view that PEP is indicated for any type of sharp injuries during contact with patients of unknown HIV status?**	**Strongly agree**	**1**
	**Agree**	**2**
	**Neutral**	**3**
	**Disagree**	**4**
	**Strongly disagree**	**5**

Study number	

	**I don't know**	**6**

Section 5: Practices regarding HIV infection control

In this section kindly answer the questions according to what you practice regarding occupational HIV exposure and usage of HIV PEP.

**5a. Do you use personal protective equipment when anticipating contact with patient blood and body fluid?**	**Yes**	**1**
	**No**	**2**
**5b. Under what circumstances do you dispose needles and sharp objects into dedicated biohazard bins?**	**Everytime**	**1**
	**When used on an HIV positive patient**	**2**
**5c. Do you recap needles?**	**Yes, if not used on a patient**	**1**
	**Never**	**2**
**5d. When do you seal the sharps disposal bins?**	**When ¾ full**	**1**
	**When half full**	**2**
	**When completely full**	**3**

You have come to the end of this questionnaire. Your help in filling out and completing this questionnaire is highly appreciated.
