# Supplementary material for: Occupational exposure to HIV among nurses at a major tertiary hospital: Reporting and utilization of post-exposure prophylaxis; A cross-sectional study in the Western Cape, South Africa
Source: PLoS One. 2020 Apr 14;15(4):e0230075. doi: 10.1371/journal.pone.0230075 (PMC7156052; doi:10.1371/journal.pone.0230075)

PLOS ONE

Occupational exposure to HIV among nurses at a major tertiary hospital: Reporting and utilization of post exposure prophylaxis; A cross-sectional study in the Western Cape, South Africa

--Manuscript Draft--

Manuscript Number:	PONE-D-19-25121
Article Type:	Research Article
Full Title:	Occupational exposure to HIV among nurses at a major tertiary hospital: Reporting and utilization of post exposure prophylaxis; A cross-sectional study in the Western Cape, South Africa
Short Title:	Occupational exposure to HIV and utilization of PEP among nurses at a major tertiary hospital in South Africa
Corresponding Author:	Katlego Tebogo Kabotho, MSc<br>Stellenbosch University Faculty of Medicine and Health Sciences<br>Cape Town, Western Cape SOUTH AFRICA
Keywords:	Occupational HIV, health care workers, knowledge, attitudes, practices , post exposure prophylaxis, Western Cape, South Africa.
Abstract:	While treatment for HIV has greatly improved patient outcomes, health care workers, including nurses, remain at high risk of occupational exposure. The risk of exposure is a continuous concern in the South African health system that is overburdened by multiple stressors, including the highest HIV caseload in the world. The aim of this study was to estimate the prevalence of occupational exposure to HIV, reporting and utilization of post exposure prophylaxis, knowledge, attitudes towards HIV post exposure prophylaxis and infection control practices among nurses at a tertiary hospital in the Western Cape, South Africa.<br>Methods<br>A cross-sectional study was conducted at Tygerberg hospital in January 2019. Participants were front line nurses working in randomly selected wards. A self-administered questionnaire was used to collect data from participants.<br>Results<br>Of the 160 participants who took part in the survey, 17 had occupational exposure to HIV (prevalence 10.6%, 95% CI 6.7 – 16.6), and of the 17 exposed, 10(58.8%) reported needle stick injuries. From those who were exposed, only 10 (58.8%) reported the incidents and went on post exposure prophylaxis. However, only 6 out of the 10 completed their treatment. Half (50%) of the participants had inadequate knowledge on HIV post exposure prophylaxis, 83.3% had adequate attitudes towards HIV post exposure prophylaxis and 75% had adequate infection control practices.<br>Conclusion<br>One out of every nine nurses had occupational exposure to HIV at a major tertiary hospital with poor reporting and utilization of post exposure prophylaxis. The high proportion of needle stick injuries highlights the need for better infection control training. Similarly, the low levels of HIV post exposure prophylaxis knowledge shows the need for structured intervention and in-service training for health care workers.<br>Keywords<br>Occupational HIV exposure, post exposure prophylaxis, knowledge, attitudes, practices, Western Cape, South Africa.
Order of Authors:	Katlego Tebogo Kabotho, MSc
	Tawanda Chivese
Additional Information:	
Question	Response
Financial Disclosure<br><br>Enter a financial disclosure statement that describes the sources of funding for the	The authors received no specific funding for this work.

Powered by Editorial Manager® and ProduXion Manager® from Aries Systems Corporation

work included in this submission. Review the submission guidelines for detailed requirements. View published research articles from *PLOS ONE* for specific examples.<br><br>This statement is required for submission and **will appear in the published article** if the submission is accepted. Please make sure it is accurate.<br><br>**Unfunded studies**<br>Enter: *The author(s) received no specific funding for this work.*<br><br>**Funded studies**<br>Enter a statement with the following details:<br>• Initials of the authors who received each award<br>• Grant numbers awarded to each author<br>• The full name of each funder<br>• URL of each funder website<br>• Did the sponsors or funders play any role in the study design, data collection and analysis, decision to publish, or preparation of the manuscript?<br>• **NO** - Include this sentence at the end of your statement: *The funders had no role in study design, data collection and analysis, decision to publish, or preparation of the manuscript.*<br>• **YES** - Specify the role(s) played.<br><br>* typeset	
**Competing Interests**<br><br>Use the instructions below to enter a competing interest statement for this submission. On behalf of all authors, disclose any competing interests that could be perceived to bias this work—acknowledging all financial support and any other relevant financial or non-financial competing interests.<br><br>This statement **will appear in the published article** if the submission is accepted. Please make sure it is accurate. View published research articles from *PLOS ONE* for specific examples.	The authors have declared that no competing interests exist.

**NO authors have competing interests**<br><br>Enter: *The authors have declared that no competing interests exist.*<br><br>**Authors with competing interests**<br><br>Enter competing interest details beginning with this statement:<br><br>*I have read the journal's policy and the authors of this manuscript have the following competing interests: [insert competing interests here]*<br><br>* typeset	
**Ethics Statement**<br><br>Enter an ethics statement for this submission. This statement is required if the study involved:<br><br>• Human participants<br>• Human specimens or tissue<br>• Vertebrate animals or cephalopods<br>• Vertebrate embryos or tissues<br>• Field research<br><br>Write "N/A" if the submission does not require an ethics statement.<br><br>General guidance is provided below. Consult the submission guidelines for detailed instructions. **Make sure that all information entered here is included in the Methods section of the manuscript.**	The study was carried out according to the principles of the Helsinki Declaration. Ethical clearance was obtained from the medical research ethics committee in Stellenbosch University (reference number 7751) while permission to carry out the study was received from Tygerberg Hospital research committee and Western Cape Province (reference number WC_201809_012).

**Format for specific study types**<br><br>**Human Subject Research (involving human participants and/or tissue)**<br>• Give the name of the institutional review board or ethics committee that approved the study<br>• Include the approval number and/or a statement indicating approval of this research<br>• Indicate the form of consent obtained (written/oral) or the reason that consent was not obtained (e.g. the data were analyzed anonymously)<br><br>**Animal Research (involving vertebrate animals, embryos or tissues)**<br>• Provide the name of the Institutional Animal Care and Use Committee (IACUC) or other relevant ethics board that reviewed the study protocol, and indicate whether they approved this research or granted a formal waiver of ethical approval<br>• Include an approval number if one was obtained<br>• If the study involved *non-human primates*, add *additional details* about animal welfare and steps taken to ameliorate suffering<br>• If anesthesia, euthanasia, or any kind of animal sacrifice is part of the study, include briefly which substances and/or methods were applied<br><br>**Field Research**<br><br>Include the following details if this study involves the collection of plant, animal, or other materials from a natural setting:<br>• Field permit number<br>• Name of the institution or relevant body that granted permission	
**Data Availability**<br><br>Authors are required to make all data underlying the findings described fully available, without restriction, and from the time of publication. PLOS allows rare exceptions to address legal and ethical concerns. See the PLOS Data Policy and FAQ for detailed information.	Yes - all data are fully available without restriction

A Data Availability Statement describing where the data can be found is required at submission. Your answers to this question constitute the Data Availability Statement and **will be published in the article**, if accepted.<br><br>**Important:** Stating 'data available on request from the author' is not sufficient. If your data are only available upon request, select 'No' for the first question and explain your exceptional situation in the text box.<br><br>Do the authors confirm that all data underlying the findings described in their manuscript are fully available without restriction?	
**Describe where the data may be found in full sentences. If you are copying our sample text, replace any instances of XXX with the appropriate details.**<br><br>• If the data are **held or will be held in a public repository**, include URLs, accession numbers or DOIs. If this information will only be available after acceptance, indicate this by ticking the box below. For example: *All XXX files are available from the XXX database (accession number(s) XXX, XXX.).*<br>• If the data are all contained **within the manuscript and/or Supporting Information files**, enter the following: *All relevant data are within the manuscript and its Supporting Information files.*<br>• If neither of these applies but you are able to provide **details of access elsewhere**, with or without limitations, please do so. For example:<br><br>*Data cannot be shared publicly because of [XXX]. Data are available from the XXX Institutional Data Access / Ethics Committee (contact via XXX) for researchers who meet the criteria for access to confidential data.*<br><br>*The data underlying the results presented in the study are available from (include the name of the third party*	N/A

and contact information or URL).<br>• This text is appropriate if the data are owned by a third party and authors do not have permission to share the data.<br><br>* typeset	
Additional data availability information:	

Powered by Editorial Manager® and ProduXion Manager® from Aries Systems Corporation

Manuscript

Click here to download Manuscript FINAL THESIS DOCUMENT 20484909-2019.docxPLOS ONE.docx

Manuscript

Title: Occupational exposure to HIV among nurses at a major tertiary hospital: Reporting and utilization of post exposure prophylaxis; A cross-sectional study in the Western Cape, South Africa

Katlego T Kabotho*[1], Tawanda Chivese[1,2,3]

1. Department of Global Health Centre for Evidence Based Health Care, Division of Epidemiology and Biostatistics, Faculty of Medicine and Health Sciences, University of Stellenbosch, Cape Town, South Africa. Email: kabothokatlego@yahoo.com

*Corresponding author

2. Chronic Disease Initiative for Africa, Department of Medicine, Faculty of Medicine and Health Sciences, University of Cape Town, Cape Town, South Africa,

3. SAMRC/Wits Developmental Pathways for Health Research Unit, Department of Paediatrics, Faculty of Medicine and Health Sciences, University of the Witwatersrand, Johannesburg, South Africa. Email: tchivese@sun.ac.za

Abstract

While treatment for HIV has greatly improved patient outcomes, health care workers, including nurses, remain at high risk of occupational exposure. The risk of exposure is a continuous concern in the South African health system that is overburdened by multiple stressors, including the highest HIV caseload in the world. The aim of this study was to estimate the prevalence of occupational exposure to HIV, reporting and utilization of post exposure prophylaxis, knowledge, attitudes towards HIV post exposure prophylaxis and infection control practices amongst nurses at a tertiary hospital in the Western Cape, South Africa.

Methods

A cross-sectional study was conducted at Tygerberg hospital in January 2019. Participants were front line nurses working in randomly selected wards. A self-administered questionnaire was used to collect data from participants.

Results

Of the 160 participants who took part in the survey, 17 had occupational exposure to HIV (prevalence 10.6%, 95% CI 6.7 – 16.6), and of the 17 exposed, 10(58.8%) reported needle stick injuries. From those who were exposed, only 10 (58.8%) reported the incidents and went on post exposure prophylaxis. However, only 6 out of the 10 completed their treatment. Half (50%) of the participants had inadequate knowledge on HIV post exposure prophylaxis, 83.3% had adequate attitudes towards HIV post exposure prophylaxis and 75% had adequate infection control practices.

Conclusion

One out of every nine nurses had occupational exposure to HIV at a major tertiary hospital with poor reporting and utilization of post exposure prophylaxis. The high proportion of needle stick injuries highlights the need for better infection control training. Similarly, the low levels of HIV post exposure prophylaxis knowledge shows the need for structured intervention and in-service training for health care workers.

Keywords

Occupational HIV exposure, post exposure prophylaxis, knowledge, attitudes, practices, Western Cape, South Africa.

Acronyms:

CDC: Centre of disease control

CI: Confidence interval

HCW: Health care workers

HIV: Human immunodeficiency virus

NSI: Needle stick injury

PEP: Post- exposure prophylaxis

PI: Percutaneous injuries

WHO: World Health Organization

Background

HIV infections continue to be on the rise worldwide despite different strategies and policies that have been implemented over the years. Recent statistics show that globally, 37.9 million people are currently living with HIV while 1.7 million were recently infected with HIV during the year 2018(1). Due to the rise in HIV infections, there is increasing contact of healthcare workers (HCW) with people living with HIV. HCWs are at risk of exposure to HIV infected material during their work, especially in sub-Saharan Africa, where the majority of people living with HIV reside. South Africa has the highest number of people living with HIV, currently estimated at 7.7 million (1). Those at a higher risk of exposure to HIV, including HCWs must be equipped with knowledge in order to prevent infection (2).

The World Health Organisation (WHO) provides recommendations that guide health care professionals in the event of an exposure to HIV in the work place (3). In South Africa, post exposure prophylaxis (PEP) is widely available especially for HCWs (4). Nonetheless, HCWs must have adequate information about HIV PEP as this will inform their action post exposure. Evidence from several studies has shown that the overall level of knowledge regarding HIV PEP is generally inadequate among healthcare workers (5, 6 ,7). This is despite reported positive perceptions regarding the treatment (8). Determinates for poor uptake of HIV PEP by those exposed are not very well known, although these may include poor knowledge, poor attitudes and lack of clear guidelines and reporting pathways.

In South Africa, knowledge and practices to protect HCWs from blood borne diseases remain inadequate in low resource settings (9). This may be due to lack of training and inadequate exposure to relevant information on HIV PEP. In Cameroon 80 nurses were

surveyed to assess their knowledge and practices on HIV PEP. In all, 73.7% of the participants had poor knowledge about HIV PEP (10). Although the majority in the previously mentioned study (83.8%), had heard about PEP, only 10 (12.5%) received formal training on PEP. This shows a gap in HIV PEP training in low-resource facilities.

Reviewed literature has shown that nurses are the most affected by occupational exposure to HIV compared to other cadres in the health sector. In 2016, a study in Ethiopia indicated nurses, apart from medical doctors, were the most affected by exposures 58.2%, compared to 30.8% laboratory and 23.3% other professions(11). In another survey in South West Ethiopia, higher rates of percutaneous injuries were observed among midwives and nurses (91.7% and 81%), compared to doctors 77.8% (12).

In 2017, a systematic review and meta-analysis with 65 cross sectional studies from 21 African countries, found high pooled lifetime and 12-month prevalence of occupational exposure to body fluids of 65.7% and 48.0%, respectively (13). Exposure was largely due to percutaneous injury with estimated 12-month prevalence of 36.0% (95% CI: 31.2–40.8). In 2016, a study in Botswana revealed that 26% participants had been exposed to sharps injuries or splashed with fluids 3 months prior to the survey (14). Of these, only 160 (37%) reported the exposure to the relevant persons and 67% of these went on to take HIV PEP treatment, however only 71% of them completed their medication.

In South Africa, a few studies have investigated exposure to HIV among HCWs, although data is lacking from the major tertiary hospitals. During 2008, 42 out of 53 (79.2%) intern doctors reported exposure to blood or body fluids of which 64% were percutaneous injuries and 36% mucosal (15). Similarly, in 219 at the Far East Rand Hospital in the Gauteng

Province, 136 out of 175 (77.7%) reported exposure to blood and body fluids among interning doctors (16). To our knowledge, there are no recent studies published on nurses' exposure to HIV at the workplace in South Africa. Further, in the Western Cape, the prevalence of occupational exposure to HIV among HCWs is not known, as there are no recently published studies, to our knowledge.

The main objective of this study was to estimate the prevalence of occupational HIV exposure, reporting and utilization of PEP in nurses at a major referral hospital in the Western Cape of South Africa. Further, the research aimed to estimate the nurses' knowledge and attitudes towards HIV PEP as well as assess their practices towards infection control. A secondary aim of the research was to investigate associations between demographic and professional characteristics of the nurses and risk of occupational exposure to HIV.

Methods

Study design and setting

A cross sectional study was conducted at Tygerberg Hospital. This is the largest hospital in the Western Cape and the second largest hospital in South Africa with an estimated number of eight thousand staff members and about two thousand nurses. Tygerberg hospital has more than 1384 beds and offers 28 specialist services.

Study population

The study population consisted of nurses working at the facility. All consenting nurses who worked directly with patients were included in the study. Nurses in management and administrative positions were excluded from taking part in the study as their work does not usually involve direct contact with patients or possibly infectious material.

Statistical considerations

Sample size determination

The sample size was determined using an acceptable margin of error of 7%, 95% confidence level and a single population proportion formula considering a prevalence of 60%, most studies reported a prevalence between 40 – 80%. A survey done in Ethiopia (11) and Nigeria (17) reported a prevalence rate of 46% and 67.5% respectively, while in South Africa two studies (15, 16) revealed a prevalence of 79.2% and 77.7% respectively.

The total required sample size was calculated using the formula below:

$$N = \frac{1.96^2 \text{ x } (1-p)^2}{d^2}$$

- where p =0.6, d =0.07

The overall estimated sample size was therefore 126. To ensure we reached the required sample size while taking into consideration possible 20% non-response (due to declines, absenteeism, and lack of time for participation) a sample size of 152 participants was required.

Sampling technique

A two-stage cluster random sampling using *Stata 15 statistical software* was used to select participants for the survey. Ten departments were randomly selected out of an overall 24 departments, followed by random selection of 2 wards from each of the 10 departments. All the nurses in the selected wards were invited to participate in the study.

Data collection

A self-administered questionnaire was used to collect data (Appendix 1). The questionnaire was developed after reviewing different literature (8, 18). A pilot study was done to test the procedure and to determine relevance of questions on 20 intern medical students from the same hospital.

We made a few amendments to each section of the questionnaire after the plot study. In section 1 of demographics, we changed the option, “caucasian” to “white” in the question “which race do you identify yourself as?” Section 2 was about occupational exposure to HIV, in question 2a we added the instruction “if the answer is no, please proceed forward to section 3 of the questionnaire” we also added question 2g, “did you start on treatment?”. A few questions were dropped in section 3 (which was about knowledge on HIV PEP) because of their repetitive nature in section 3. The question, “have you attended any training on HIV PEP?” was removed because it appeared in section 1. Question 3f, “under which circumstances would you not take HIV PEP?” was also removed because it was similar to 3d. Some questions were amended to add options from which the participants were to choose their answers, for instance the option, “I don’t know”, was added to section 3 and 4 (attitudes towards HIV PEP) of the questionnaires for those who did not know the answer to

the question. In section 5, which is about HIV infection control practices, we dropped the question, “what would prompt you to take PEP?”, because it was similar to a question asked in section 3.

After informed consent (Appendix 2) was obtained, the questionnaires were distributed to all the nurses in the ward at the time. To minimize response bias due to night staff not participating, questionnaires for the night staff were left with the nurse in charge for them to complete. These questionnaires were collected the next morning.

Data extraction and statistical analysis

Data were captured into *Microsoft Office Excel* and exported to *STATA 2015* for analysis. Where numerical data were normally distributed, means and standard deviations were used to describe the data. Where data were not normally distributed, the data were described by medians and interquartile range (IQR). For categorical data (for example, gender and marital status) frequencies and percentages were reported.

Determination of occupational HIV exposure

The overall prevalence of occupational HIV exposure was calculated from the number of self- reported incidents relative to the total number of study participants.

Determination of knowledge

Four questions were asked to assess the knowledge of the participants on HIV PEP. The overall knowledge score was computed by adding up all the correct knowledge answers and divided by the total number of questions asked, then expressed as a percentage. A median

(IQR) knowledge percentage score was reported. The percentage score was categorised as <50% poor, 50-74% good and >75% as adequate knowledge.

Determination of attitudes

Attitudes were determined using a 5-point Likert scale. Responses ranged from strongly agree to strongly disagree. However, there was an option for those who did not know the answer to the question to state so. All the participants who selected "I don't know" were not included in the analysis therefore, the responses "don't know" were treated as missing data. To get the overall attitudes mark, all 6 questions were scored individually, for questions where the preferred responses were "strongly agree/agree" or "strongly disagree/disagree" a mark of 1 was given and a zero otherwise. We then added all the individual scores and expressed it as a percentage. Median and IQR were reported. Further, attitude scores were categorised as <50% poor, 50-74% good and >75% as adequate attitudes (19). Frequencies and percentages for each question were also tabulated.

Determination of practices

Four questions were asked to assess the practices of participants regarding infection control. The overall practice score was computed by adding up all the correct practices per question and divided by the total, and then expressed as a percentage. A median (IQR) practice percentage score was reported. The percentage score was categorised as <50% poor, 50-74% good and >75% as adequate practice (19).

Statistical analysis

Bivariate analysis

For categorical data, Chi-squared or Fisher exact test were used to measure the association amongst variables. Independent and dependent variables were associated with each other to determine the ones with significant association. A p-value of 0.05 was considered statistically significant. The 95 % confidence intervals (95%CI) were reported for estimates, where appropriate.

Investigation of factors associated with exposure

A multiple variable logistic regression was used to investigate determinants of occupational exposure to HIV. Occupational exposure to HIV was treated as binary outcome while the predictor variables were the practice score, professional training, experience and demographic variables.

Ethics

The study was carried out according to the principles of the Helsinki Declaration (20). Ethical clearance was obtained from the medical research ethics committee in Stellenbosch University (reference number 7751) (Appendix 3) while permission to carry out the study was received from Tygerberg Hospital research committee (Appendix 4) and Western Cape Province (reference number WC_201809_012).

Results

Sociodemographic characteristics of participants

A total of 168 nurses were approached to take part in the study and, of these, 160 agreed to fill out the questionnaire. Eight participants did not consent to take part in the study, resulting in a 95.24 % response rate. One participant did not complete the demographic section of the questionnaire. Most of the participants, 147 (92.45%) were female. The mean age (SD) of the participants was 40.63 years (SD 9.91). Only 40 (25.16%) of the 160 participants had attended formal training on HIV PEP. A summary of the demographics by exposure status is shown in Table 1.

Table 1. Socio-demographics of participants

Variable		Overall	Exposed	Not exposed	P value
Gender	Females, n (%)	147 (92.45)	14 (9.52)	133 (90.48)	
	Males, n (%)	12 (7.55)	3 (25)	9 (75)	0.099
Race	Black, n (%)	73 (45.91)	3 (4.11)	70 (95.89)	
	White, n (%)	4 (2.52)	0	4 (100)	0.013
	Mixed ancestry, n (%)	81 (50.94)	14 (17.28)	67 (82.72)	
Marital status	Single, n (%)	68 (43.40)	7 (10.29)	61 (89.71)	
	Married, n (%)	70 (44.03)	8 (10.43)	62 (88.57)	0.902
	Divorced, n (%)	15 (9.43)	1 (6.67)	14 (93.33)	
	Widowed, n (%)	5 (3.14)	1 (20)	4 (80)	
Age, mean (SD) years		40.63 (9.91)	45(34 – 49)	41 (34 – 48)	0.563
Years of practice, median (IQR)		9 (4-24)	15 (6 – 25)	8.5(4 – 24)	0.254
Education, n (%)	diploma	126 (79.25)	12 (9.52)	113 (90.48)	
	bachelor	20 (12.58)	4 (20)	16 (80)	
	Masters	1 (0.63)	0	1 (100)	0.433
	other	12 (7.55)	1 (8.33)	11 (91.67)	

Training attendance on HIV PEP, n (%)	yes	40 (25.16)	3 (7.5)	37(92.5)	0.443

Frequency and reporting of occupational exposure to HIV, and PEP utilization

Amongst the 160 participants who took part in the study, 17 of the respondents got occupationally exposed to HIV in the past 12 months of their work, resulting in an overall prevalence of 10.63% (95% CI 6.72 – 16.60%). From the 17 who were exposed, only 10 (58.82%) reported the incidents and sought treatment (Fig 1). All the 10 participants who reported exposures started on treatment. However only 6 (60.0%) of the 10 respondents finished treatment resulting in a 40.0% dropout rate. Out of the 17 who were exposed, 10 (58.82%) had needle stick injuries, 1 (5.88%) had a cut by a sharp object while 6 (35.29%) had contact with body fluids as shown in Fig 2.

Fig 1. Occupational exposure to HIV, reporting and utilisation of PEP

Fig 2. Occupational exposure to HIV classification

Knowledge of participants about HIV PEP

Out of 159 respondents with complete data, 115 (72.33%) of the participants responded that they had heard about HIV PEP. From these 115 respondents, 57 (49.6%) named training as the source of awareness. Participants correctly answered questions 3, 4, 5 as follows; 70 (44.03), 93 (58.49%), 66 (41.51%) respectively (Table 2). The overall median knowledge score was 50% (IQR 25-75). When the knowledge score was categorised, only 46.54% had "adequate" knowledge while 32.08% had "poor" knowledge of HIV PEP. There were no

significant differences, between participants who had occupational exposure to HIV and those not exposed, in either the knowledge percent score or categorized knowledge percent score (Table 3).

Table 2. Knowledge regarding HIV PEP

Question	Answer	N (%)
Have you heard about PEP?	Yes	115 (72.33)
When do you think HIV PEP should not be administered if exposed?	When source is HIV negative?	70 (44.03)
	When patient is known to be HIV positive	20(12.58)
	When HIV status of source is unknown	16 (10.06)
	I don't know	52 (32.70)
When is the recommended time to take PEP?	Any time after exposure, does not matter when	36 (22.64)
	Within 72 hours of exposure	93 (58.49)
	I don't know	30(18.87)
What is the length of time for taking PEP?	For 28 days	66 (41.51)
	For six months	28(17.61)
	For a life time	6 (3.77)
	I don't know	59 (37.11)

Table 3. Overall scores and categories of knowledge, attitudes and practices

		Overall	Exposed, n = 17	Unexposed, n = 143	p-value
Knowledge score percent	Median (IQR)	50 (25-75)	75 (50-75)	50 (25-75)	0.258
Knowledge score categories, n (%)	Poor	51 (32.08)	3 (17.65)	48 (33.80)	
	Good	34 (21.38)	3 (17.65)	31 (21.83)	0.258
	Adequate	74 (46.54)	11 (64.71)	63 (44.37)	
Attitude score percent	Median (IQR)	83.33 (66.67 – 88.33)	83.33 (66.67 – 88.33)	83.33 (66.67 – 88.33)	0.373

Attitude score categories, n (%)	Poor	1 (0.67)	1 (0.75)	0	
	Good	49 (32.89)	5(31.25)	88 (66.17)	0.928
	Adequate	99 (66.44)	11(68.75)	88 (66.17)	
Practice score Percent	Median IQR	75 (75 – 100)	100 (75 – 100)	75 (75 – 100)	0.153
Practice score categories, n(%)	Poor	5 (3.18)	1 (5.88)	4 (2.86)	0.496
	Good	22 (14.01)	1 (5.88)	21 (15.00)	
	Adequate	130 (82.80)	15 (88.24)	115 (82.14)	

Attitudes of participants towards HIV PEP

For questions one, two and three, most of participants chose “strongly agree” or “agree” as a preferred choice (95.92%, 100% and 99.33% respectively). In question four, 85.11% selected “strongly agree” or “agree” as a response. Out of 138 respondents, 14(9.42%) and 1 (0.72%) chose “disagree” and “strongly disagree” respectively, in question 5. In question six, 79 (57.25%) and 45(32.61) chose the answers “strongly agree” and “agree” respectively (Table 4). The majority of participants had adequate attitudes towards HIV PEP, the median score was 83.33 % (IQR 66.67-83.33). There were no significant differences, between participants who had occupational exposure to HIV and those not exposed, in either the attitude percent score or categorized attitude percent score (Table 3).

Table 4. Attitudes of participants towards HIV PEP

Response	Strongly agree	Agree	Neutral	Disagree	Strongly disagree
Do you think HIV PEP is important? n (%)	115(7.23)	26(17.69)	4(2.72)	1(0.68)	1 (0.68)
Do you believe that training on HIV PEP is important to influence people to comply with PEP guidelines? , n (%)	108(72.48)	41(27.52)	0	0	0
Do you think there should be an HIV PEP guideline poster on the walls? n (%)	113(75.33)	36(24.00)	1(0.67)	0	0
Do you believe PEP reduces likelihood of being HIV positive after exposure? n (%)	70(49.65)	51(36.17)	11(7.80)	9 (6.38)	0
Do you think that HIV PEP should be administered if the exposure is not with patient of known HIV positive status? n(%)	64(46.38)	42(31.16)	17(12.32)	14(9.42)	1 (0.72)
What is your opinion on the view that PEP is indicated for any type of sharp injuries during contact with patients of unknown HIV status? n (%)	79(57.25)	45(32.61)	12(8.70)	0	2(1.45)

Practices of participants regarding infection control

From a total of 159 respondents, 151 (94.97%) reported that they used personal protective equipment (PPE) whenever contact with patient blood and body fluids was anticipated. Out of 156 participants, 154 (98.72%) reported that they always discarded sharps in an appropriate container. One hundred and seventeen out of 153 (76.47%) participants reported that they never recap needles. From 153 participants who responded to question 3 “when do you seal the sharps disposal bin?” 93(60.78%) practice the right exercise (Table 5). The overall median practice score was 75% (IQR 75-100).

When the practice score was categorised, 82.8% participants had “adequate” while 3.18% had “poor” infection control practices. There were no significant differences, between participants who had occupational exposure to HIV and those not exposed, in either the practice percent score 100% (IQR 75-100) vs 75% (IQR 75-100), p = 0.153 or categorized knowledge percent score (Table 3).

Table 5. Practices towards infection control measures

Question	Answer	N (%)
**Do you use personal protective equipment when anticipating contact with patient blood and body fluid?, n(%)**	Yes	151 (94.97)
**Under what circumstances do you dispose needled and sharp objects into the dedicated biohazard bins?, n(%)**	Every time	154(98.72)
	When used on an HIV positive patient	2 (1.28)
**Do you recap needles?, n(%)**	Yes, if not used on a patient	36 (23.53)
	Never	117 (76.47)
**When do you seal the sharps disposal bin? , n(%)**	When ¾ full	93 (60.78)
	When half full	14 (9.15)
	When completely full	46 (30.07)

Exploration of factors associated with occupational exposure to HIV

After multiple variable logistic regression (Fig. 3), there was a trend towards lower risk of exposure in female nurses, compared to males (OR 0.27, 95%CI 0.05-1.37, p = 0.114) and a trend towards higher risk in nurses untrained in HIV PEP, compared to those who received formal training in PEP (OR 2.97 95% CI 0.64 – 13.83 p=0.166), although not statistically significant. There were no associations between occupational exposure to HIV and any of the other demographic characteristics as well as infection control practices scores.

Fig 3: Multiple variable logistic regression for factors associated with occupational exposure to HIV

NOTES
LCL- lower confidence level (lower 95%CI)
UCL- upper confidence level (Upper 95%CI)
OR- odds ratio

Discussion

In this study, we found that one out every nine nurses at a major tertiary hospital in the Western Cape of South Africa had occupational exposure to HIV, of which almost two-thirds was exposure due to needle stick injuries. We also found inadequate reporting of exposures to occupational health services and poor PEP completion rate. The findings show that half of the participants had inadequate knowledge of HIV PEP, although most of the participants had both adequate attitudes towards PEP and good practices towards infection control.

The current exposure rate (11%) in this study is one of the lowest compared to other literature. A survey done amongst HCWs in Ethiopia (11) and Nigeria (17) reported a prevalence rate of 46% and 67.5% respectively, while in South Africa two studies revealed a prevalence of 79.2% and 77.7% respectively (15, 16) in a cohort of inter doctors. The prevalence rate close to the one reported in this study is 19.2%, from a study carried out amongst HCWs in Uganda (21). In this study 65% of the exposures were attributable to percutaneous injuries with most of them due to needle stick injuries (58.8%). In the year 2015 in Tanzania, a report showed a 62.9% exposure to needle stick injuries (5). Similarly an exposure rate of 63.6% was reported from a study done in Nigeria in 2011 (17). In most studies needle stick injuries account for most of the exposures and our findings follow the same trend.

Only 59% of the participants reported the exposure incidents. There were several reasons. A lack of clear knowledge on what to do after the exposure was frequently the case in our study. One participant indicated that after exposure she did not report the case to the relevant authorities as she was told by a fellow colleague that she would have to go on HIV PEP treatment for 6 months. Another participant revealed that a colleague of hers' told her "not to worry about the exposure" as it was "just a blood splash" so she did not report it. In South East Ethiopia, 59% of participants did not report injuries due to the following reasons; time constraints, sharps which caused injury were not used on any patient, the source patients did not have disease of concern, and lack of knowledge that it should be reported (6). This may have serious implications as undocumented exposure injuries could prevent injured HCWs from receiving PEP therefore potentially resulting in new HIV infections. Another concern is that 40% of participants did not finish PEP. Our findings are comparable to those of a study done Botswana in 2016, where 69% of participants received PEP and only 71% completed their medication (14). In this study, two participants discontinued treatment due to side effects from medication. Although our study is small, and the exposed numbers were also few, the lack of completion of PEP is a clinical and public health problem which warrants attention. Bigger studies may be needed to explore this and to find out the reasons why HCWs are not completing PEP, despite advancement in ARV formulations, which are now less toxic.

In this study 50% of the participants had inadequate knowledge regarding HIV PEP. These findings are consistent with data from similar studies in Africa. In the year 2015, Cameroon had 74% of the participants with inadequate knowledge about HIV PEP(10) whereas 37% was reported in North East Ethiopia. The overall attitudes of the participants towards HIV

PEP was positive with a median score of 83% similar to findings from Botswana in 2019 (18), where participants had a mean score of 82.2% positive attitudes towards PEP. The participants had adequate practices of 75% overall. However, one of the respondents’ who experienced a needle stick injury reported being pricked by a needle that was wrapped in a small opaque plastic bag as she was cleaning the area. This shows an improvement is still required in infection control practice. The finding that 30% of respondents seal the sharps container when full, is a concerning result as a sharps container should never be filled up to the “full” mark. One participant mentioned that often when it’s filled to the top and cannot close, they physically remove the needled or force the bin to close. The potential for injury and HIV infection from these actions cannot be understated. There seems to be a gap in infection control training and more training may be required.

One limitation of the study was that we relied on subjective recall of participants, which may have resulted in either underreporting or over reporting of exposures. Participants could take the questionnaire home if they needed more time. We left questionnaires for night staff to complete but there was no way to check how many declined. The KAP section could have been improved by adding more questions. Lastly, our study was not powered up to detect factors associated with risk of exposure as it was a minor objective. Future studies, with bigger numbers, are needed to further explore our findings.

Conclusion

One out of every nine nurses are exposed to occupational HIV, with a worrisome proportion due to needle stick injuries, amid poor reporting of exposures and poor utilization of PEP. Despite overall acceptable attitudes towards PEP and good practices in infection control, half of the nurses at this tertiary level do not have good knowledge of HIV PEP.

Acknowledgements

I would like to thank the Lord, my heavenly Father for the grace, wisdom, strength and courage He has afforded me to complete this thesis. I would also wish to sincerely thank my supervisor Mr Tawanda Chivese for his guidance, support and contribution to this thesis. My gratitude also goes to all the nurses that took part in the survey as well Mr Bukasa who is a nurse’s manager in orthopaedic surgery ward in Tygerberg hospital for helping out in the data collection process. My thanks go to Ms Lindiwe J. Modise who assisted in proof reading the manuscript several times. To anyone else who has helped me, I am truly appreciative.

References

1. Sheet F, Update GA, Hiv N, Hiv N. 2018 GLOBAL HIV STATISTICS. 2019;1–6.

2. Mashoto KO, Mubyazi GM, Mushi AK. Knowledge of occupational exposure to HIV: a cross sectional study of healthcare workers in Tumbi and Dodoma hospitals, Tanzania. BMC Health Serv Res [Internet]. 2015;15:29. Available from: http://www.pubmedcentral.nih.gov/articlerender.fcgi?artid=4307904&tool=pmcentrez&rendertype=abstract

3. Ford N, Mayer KH, Health W, Postexposure O, Guideline P. World Health Organization Guidelines on Postexposure Prophylaxis for HIV : Recommendations for a Public Health Approach. 2015;60(Suppl 3):161–4.

4. Conradie F, Howell P. Guideline on the management of occupational and non-occupational exposure to the human immunodeficiency virus and recommendations for post-exposure prophylaxis : 2015 Update. 2015;12(2):1–14.

5. Mponela MJ, Oleribe OO, Abade A, Kwesigabo G. Post exposure prophylaxis following occupational exposure to HIV: A survey of health care workers in Mbeya, Tanzania, 2009-2010. Pan Afr Med J. 2015;21:2009–10.

6. Bekele T, Gebremariam A, Kaso M, Ahmed K. Attitude, reporting behavour and management practice of occupational needle stick and sharps injuries among hospital healthcare workers in Bale zone, Southeast Ethiopia: A cross-sectional study. J Occup Med Toxicol [Internet]. Journal of Occupational Medicine and Toxicology; 2015;10(1):1–7. Available from: http://dx.doi.org/10.1186/s12995-015-0085-2

7. Ajibola S, Akinbami A, Elikwu C, Odesanya M, Uche E. Knowledge, Attitude and practices of HIV post exposure prophylaxis amongst health workers in Lagos University teaching hospital. Pan Afr Med J. 2014;19:1–8.

8. Access O. Assessment of knowledge , attitude and practice towards post exposure prophylaxis for HIV among health care workers in Gondar , North West Ethiopia. 2013;

9. Nkoko L, Spiegel J, Rau A, Parent S, Yassi A. Reducing the Risks to Health Care

Workers from Blood and Body Fluid Exposure in a Small Rural Hospital in Thabo-Mofutsanyana, South Africa. Workplace Health Saf [Internet]. 2014;62(9):382–8. Available from: http://journals.sagepub.com/doi/10.3928/21650799-20140815-03

10. Aminde LN, Takah NF, Dzudie A, Bonko NM, Awungafac G, Teno D, et al. Occupational post-exposure prophylaxis (PEP) against human immunodeficiency virus (HIV) infection in a health district in Cameroon: Assessment of the knowledge and practices of nurses. PLoS One. 2015;10(4):1–12.

11. Kaweti G, Abegaz T. Prevalence of percutaneous injuries and associated factors among health care workers in Hawassa referral and adare District hospitals, Hawassa, Ethiopia, January 2014. BMC Public Health [Internet]. BMC Public Health; 2016;16(1):1–7. Available from: http://dx.doi.org/10.1186/s12889-015-2642-0

12. Mekonnin T, Tsegaye A, Berihun A, Kassachew H, Sileshi A. Medical Safety & Global Health Occupational Exposure to Blood and Body Fluids among Health Care Workers in Mizan Tepi University Teaching Hospital , Bench Maji Zone , South West. 2018;7(2).

13. Auta A, Adewuyi EO, Tor-Anyiin A, Aziz D, Ogbole E, Ogbonna BO, et al. Health-care workers' occupational exposures to body fluids in 21 countries in Africa: systematic review and meta-analysis. Bull World Heal Organ [Internet]. 2017;95(October):831–41. Available from: http://dx.doi.org/10.2471/BLT.17.195735

14. Kassa G, Selenic D, Lahuerta M, Gaolathe T, Liu Y, Letang G, et al. Occupational exposure to bloodborne pathogens among health care workers in Botswana: Reporting and utilization of postexposure prophylaxis. Am J Infect Control. 2016;

15. Hospital A. Occupational exposure to blood-borne or body fluid pathogens among medical interns at Addington Hospital , Durban. 2011;53(5):2003–7.

16. Aigbodion SJ, Motara F, Laher AE, Rand FE, Africa S. Occupational blood and body fluid exposures and human immunodeficiency virus post-exposure prophylaxis amongst intern doctors. :1–6.

17. Nwankwo TO, Aniebue UU. Percutaneous injuries and accidental blood exposure in surgical residents: Awareness and use of prophylaxis in relation to HIV. Niger J Clin

Pract. 2011;14(1):34–7.

18. Hospital M. Assessment of knowledge, attitudes and practices of HIV post exposure prophylaxis among the doctors and nurses in Princess Marina Hospital, Gaborone: a cross-sectional study. 2018;8688:1–11.

19. Eticha EM. Knowledge , Attitude , and Practice of Postexposure Prophylaxis against HIV Infection among Healthcare Workers in Hiwot Fana Specialized University Hospital , Eastern Ethiopia. 2019;2019.

20. Kong H, West S, States U. THE WORLD MEDICAL ASSOCIATION , INC . DECLARATION OF HELSINKI Ethical Principles for Medical Research Involving Human Subjects. 2008;(June 1964):1–5.

21. Kumakech E, Achora S, Berggren V, Bajunirwe F. Occupational Exposure to HIV: A Conflict Situation for Healthcare Workers. Int Nurs Rev. 2011;58(4):454–62.

Figure 1

Click here to download Figure Fig1.tif

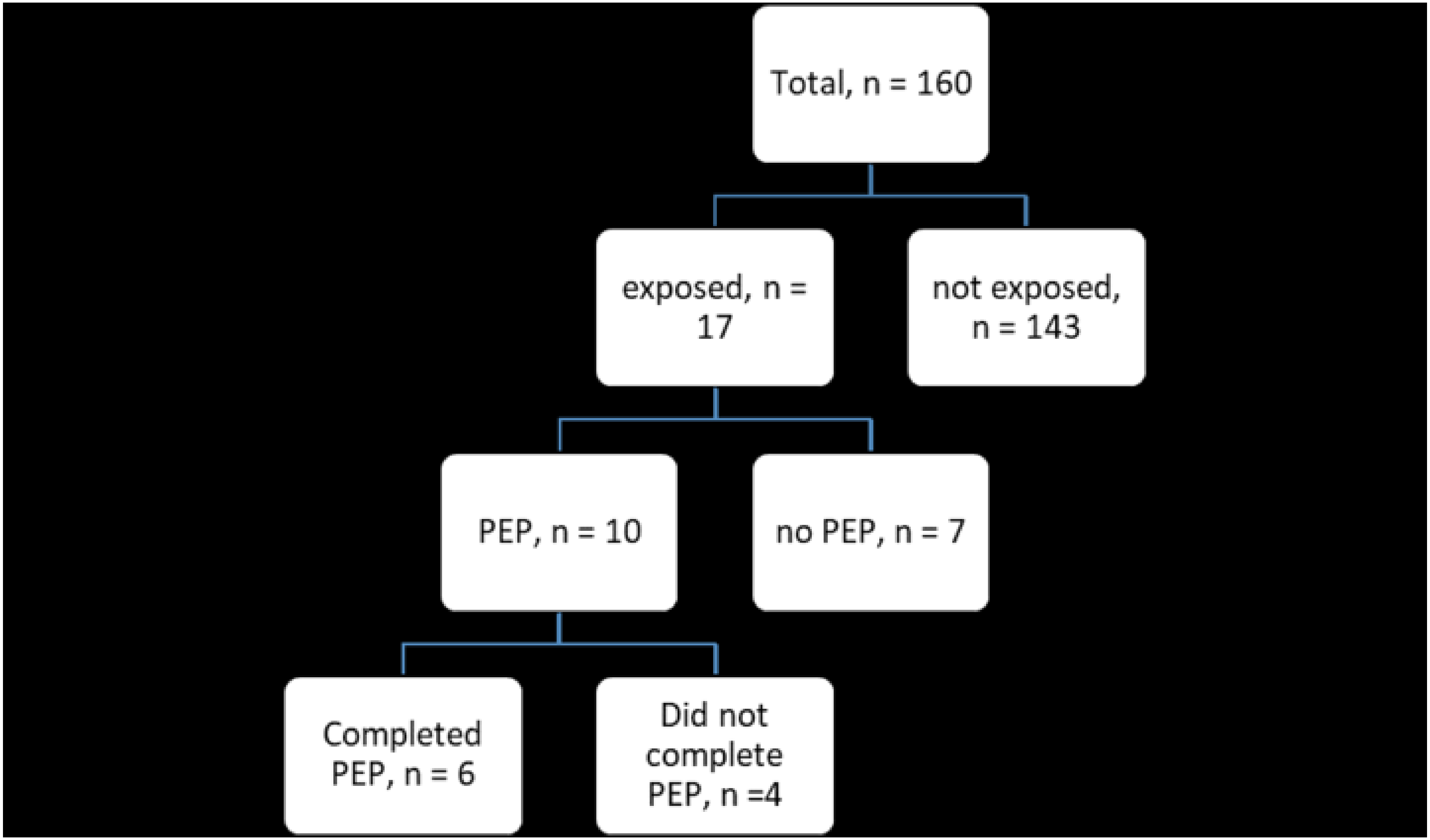

Figure 2

Click here to download Figure Fig2.tif

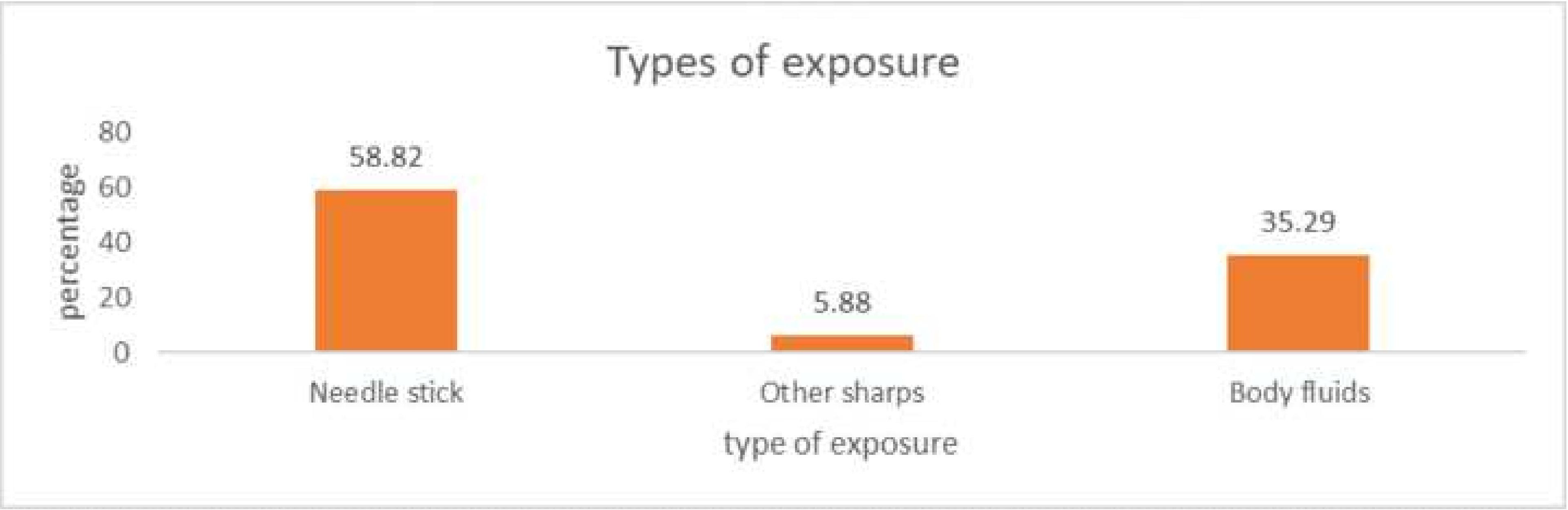

Figure 3

Click here to download Figure Fig 3.tif

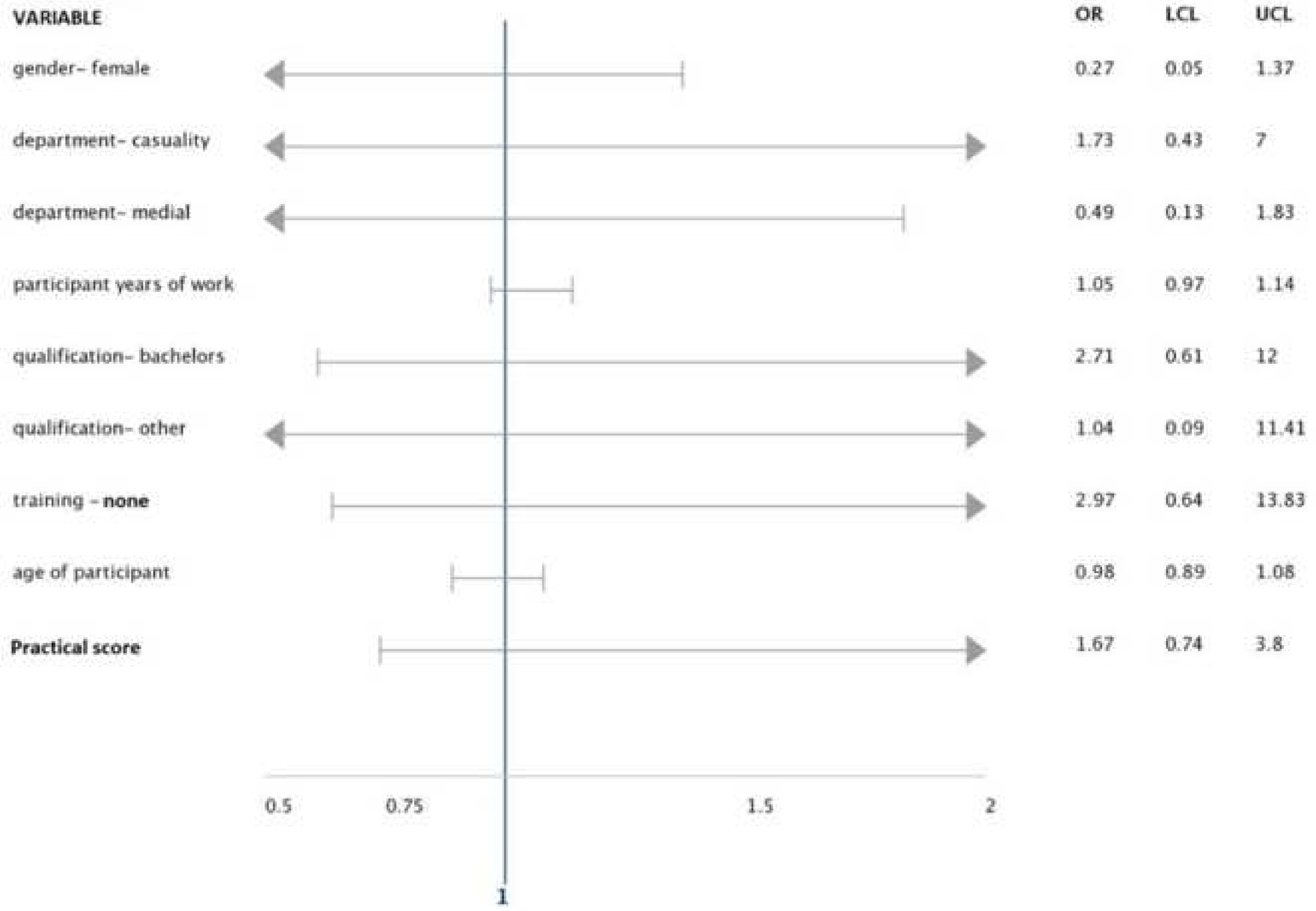

Supplement: Attachment — Submitted filename: PONE-D-19-25121_reviewer.pdf [file pone.0230075.s003.pdf]
