# Supplementary material for: Occupational exposure to HIV among nurses at a major tertiary hospital: Reporting and utilization of post-exposure prophylaxis; A cross-sectional study in the Western Cape, South Africa
Source: PLoS One. 2020 Apr 14;15(4):e0230075. doi: 10.1371/journal.pone.0230075 (PMC7156052; doi:10.1371/journal.pone.0230075)
Supplement: Attachment — Submitted filename: PLOSONE_Response_to_reviewers 30 Jan.pdf [file pone.0230075.s004.pdf]

Occupational exposure to HIV among nurses at a major tertiary hospital: Reporting and utilization of post exposure prophylaxis; A cross-sectional study in the Western Cape, South Africa

Response to reviewers

Reviewer	Response	Page/ paragraph where change affected
Is the manuscript presented in an intelligible fashion and written in standard English?	Thank you for your feedback. We made improvements to the manuscript using input on draft and are submitting a revised manuscript	Throughout the manuscript
Background. Line 86-87 “To our knowledge, there are no recent studies published on nurses’ exposure to HIV at the workplace in South Africa.” Perhaps include the following study done in Limpopo: Makhado, L., Davhana-Maselesele, M. Knowledge and uptake of occupational post-exposure prophylaxis amongst nurses caring for people living with HIV. Curationis. 2016, 39(1), a1593. http://dx.doi.org/10.4102/ curationis.v39i1.1593	Thank you for your feedback. We have cited this study in the Background.	**Background line 88-92**
Sampling and analysis: Was clustering considered in the analysis strategy (regression model)?	Thank you for your feedback. Clustering was not included in the analysis as the study was mainly exploratory. We have included this limitation in the Discussion section	**Discussion section line 371 - 372**
Data collection: How reliable is the knowledge score if participants did not complete this under supervision? Indicate the date of data collection (months and year).	Thank you for your feedback. We have added under the discussion, the limitations due to the fact that some participants were given the questionnaire to complete at home and not under supervision.<br><br>The data were collected over a period of two weeks	**Discussion section line 372-374**<br><br>**Methods section line 160-161**

	from the 4th to the 16th February 2019. This has been added to the methods	
Questionnaire validity reliability: Describe the questionnaire validity and reliability, for example, was content validity of the questionnaire checked by experts in the field? Was a Cronbach alpha calculated for the attitudes scale?	Thank you for your feedback. The questionnaire was adopted from existing studies, however some questions that were added and some removed, and a pilot study was done. We did not calculate a Cronbach alpha for the attitudes scale.<br><br>After the comment from the reviewer, we calculated the Cronbach alpha, which was 0.54. This has been added to the methods and as a limitation to the discussion.	**Methods section line 136-139**<br><br>**Method section line 139**<br><br>**Discussion section, lines 374-377**
Results: Education of nurses – did the authors distinguish between the different levels of nurses, for example assistant nurses (auxiliaries), enrolled nurses and professional nurses?	Thank you for your feedback. There was no distinguishing between levels of nurses in the study. We have added this as a limitation under the Discussion section.	**Discussion section line 371-372**
Please provide additional details regarding participant consent. In the ethics statement in the Methods and online submission information, please ensure that you have specified what type of consent you obtained (for instance, written or verbal, and if verbal, how it was documented and witnessed).	Thank you for your feedback. We obtained written consent from the participants. This has been added in the methods section and on the online submission system.	**Methods section line 153-154**
Please include additional information regarding the survey or questionnaire used in the study and ensure that you have provided sufficient details that others could replicate the analyses. For instance, if you developed a questionnaire as part of this study and it is not under a copyright more restrictive than CC-BY, please include a copy, in both the original language and English, as Supporting Information. Additionally,	Thank you for your feedback, we have included the questionnaire as part of additional information. It is in English. We have also stated when participants were recruited into the study.	**Methods section lines 109-110**

please state when participants were recruited for this study.		
Please remove ‘I would like to thank the Lord, my heavenly Father for the grace, wisdom, strength and courage He has afforded me to complete this thesis’ from the Acknowledgements section of this manuscript	Thank you for the feedback, this statement has been removed from the acknowledgements section	**Acknowledgements section line 386-389**
Data Availability statement, you have not specified where the minimal data set underlying the results described in your manuscript can be found. PLOS defines a study's minimal data set as the underlying data used to reach the conclusions drawn in the manuscript and any additional data required to replicate the reported study findings in their entirety. All PLOS journals require that the minimal data set be made fully available. For more information about our data policy, please see http://journals.plos.org/plosone/s/data-availability.	Thank you for your feedback. The data set is available and uploaded as a supplementary comma delimited data set	
